# Determinants of Beverage Consumption in Young Adults: A Multicenter Cross-Sectional Study Across Seven Major Geographic Regions of China

**DOI:** 10.3390/foods14213687

**Published:** 2025-10-29

**Authors:** Shuyi Zhou, Jianfen Zhang, Xiuhua Shen, Lina Yang, Jinsong He, Fan Zhang, Guansheng Ma, Na Zhang

**Affiliations:** 1 Department of Nutrition and Food Hygiene, School of Public Health, Peking University, Beijing 100191, Chinazhangjf@ninh.chinacdc.cn (J.Z.); 2 Department of Student Nutrition, National Institute for Nutrition and Health, Chinese Center for Disease Control and Prevention, 27 Nanwei Road, Xicheng District, Beijing 100050, China; 3 School of Medicine, Shanghai Jiao Tong University, Shanghai 200025, China; srachel@126.com; 4 School of Xiangya Public Health, Central South University, Changsha 410013, China; ylnly1997@csu.edu.cn; 5 College of Food Science and Technology, Yunnan Agricultural University, Kunming 650201, China; hejs@ynau.edu.cn; 6 School of Public Health, Hainan Medical University, No. 3 Xue Yuan Road, Longhua District, Haikou 571199, China; zhangfan@muhn.edu.cn; 7 Laboratory of Toxicological Research and Risk Assessment for Food Safety, Peking University, Beijing 100191, China

**Keywords:** beverage intake, hydration, young adults, China, determinants, NCD prevention, cross-sectional study

## Abstract

Early adulthood is a critical period for non-communicable disease (NCD) prevention, with beverage consumption being a modifiable risk factor. Evidence in young Chinese adults remains limited. We conducted a multicenter cross-sectional study (May–June 2023) of 3198 university students aged 18–25 years from seven regions of China. Beverage intake was recorded for seven days using the validated Liq.In7 diary with calibrated cups, and multivariable linear regression, including sex- and age-stratified models and interaction analyses, identified determinants. Sex, age, and sleep quality were the strongest predictors: females consumed less plain water (β = –112.75; 95% CI: –147.98 to –77.53; *p* < 0.001) and SSBs (β = –23.59; 95% CI: –34.60 to –12.57; *p* < 0.001), while plain water intake increased with age (β = 33.42; 95% CI: 19.92–46.91; *p* < 0.001). Poorer sleep quality (higher PSQI) was associated with higher SSBs consumption. SSBs intake among adults aged 22–25 years increased with higher temperature and socioeconomic tier. Interaction models confirmed that plain water intake increased with age in both sexes but remained lower in females. These findings support integrated interventions, including SSBs taxation and nutrition education, aligned with Healthy China 2030 and WHO sugar-reduction targets.

## 1. Introduction

Metabolic disorders such as obesity, type 2 diabetes (T2DM), and cardiovascular disease (CVD) remain leading causes of morbidity, premature mortality, and escalating healthcare costs worldwide [1]. According to the International Diabetes Federation’s latest Diabetes Atlas (2021), approximately 537 million adults aged 20–79 years were living with diabetes in 2021, with projections estimating 783 million by 2045, driven largely by rising incidence in low- and middle-income countries where urbanization and dietary shifts are accelerating the epidemic [2]. Beyond the human toll, these conditions account for nearly USD 1 trillion in annual health expenditures, representing a growing socioeconomic challenge [2].

China is experiencing one of the fastest nutrition transitions globally. Rapid economic development and urbanization have led to dramatic shifts toward energy-dense diets, higher sugar-sweetened beverages (SSBs) availability, and more sedentary lifestyles, coinciding with steep increases in obesity and T2DM prevalence [3]. The China Health and Nutrition Survey (CHNS) data from 1989 to 2015 reveal a dramatic increase in obesity rates from 2.9% to 16.4% among adults, with T2DM prevalence surging from 0.9% in 1980 to 11.2% in 2017 [4]. Such trends underscore the urgent need for population-level strategies to mitigate metabolic risk.

Early adulthood (18–29 years) represents a critical window for primordial prevention, as this is when long-term dietary and lifestyle patterns are established and metabolic risk factors such as elevated BMI and insulin resistance first emerge [5,6,7]. Based on the latest national survey, young adults in China (18–29 years) are among the highest consumers of sugar-sweetened beverages (SSBs), with a consumption prevalence of 20.67% and a median intake of 39.01 g/day among consumers [8]. Longitudinal studies, such as the CARDIA cohort, demonstrate that early-life metabolic abnormalities predict a two- to threefold higher lifetime risk of T2DM and CVD [9]. Intervening during this formative period may therefore prevent decades of cumulative risk and significantly reduce future healthcare costs.

Beverage consumption is an important component of diet and a modifiable risk factor for metabolic health [10]. Evidence consistently indicates that plain water, unsweetened tea [11], and coffee [12] are associated with lower risk of obesity, T2DM, and CVD [13,14], whereas habitual SSBs intake is linked to weight gain, insulin resistance, and cardiometabolic complications. For instance, a large prospective study of individuals with T2DM found that higher SSBs consumption was associated with 20% increased all-cause mortality and a 25% higher risk of incident cardiovascular disease [11], while unsweetened coffee intake was linked to a 25–30% reduction in T2DM risk in randomized controlled trials [12,15,16]. These findings highlight beverage choice as a feasible, cost-effective target for chronic disease prevention.

Beverage intake is shaped by a complex interplay of factors operating at multiple levels [17,18]. Individual factors such as age, sex, and lifestyle behaviors (e.g., sleep and physical activity), and psychosocial factor (anxiety and depression) interact with interpersonal influences, including family and peer norms, to shape beverage preferences [17,18,19]. Environmentally, hotter climates (e.g., >30 °C) boost overall fluid intake by 1–2 L/day, favoring chilled SSBs in urban China where vending machine density has risen by 40% in the past decade, according to urban planning reports [19].

Because of this multilevel complexity, effective public health strategies must go beyond individual education to include environmental and policy interventions. Fiscal measures such as SSB taxation in Mexico and the UK have achieved 10–20% reductions in purchases within two years [20,21,22]. Such successes align with the WHO Global Action Plan for the Prevention and Control of Noncommunicable Diseases [23,24] and China’s “Healthy China 2030” blueprint [25], which call for population-wide approaches to reduce added sugar intake and promote healthier beverage options.

However, evidence from China, particularly among young adults, remains limited. Most existing studies have focused on children or the general population, rarely analyzing young adults as a distinct group, despite national surveys showing that 18–29-year-olds have one of the highest SSB intakes [8]. Moreover, beverage intake is often assessed using one- to three-day 24 h dietary recalls, which may not fully capture habitual patterns due to high day-to-day variability [26]. Few studies have comprehensively examined beverage consumption through validated, multi-day assessment tools or simultaneously considered multilevel determinants, spanning individual, interpersonal, and environmental factors, within a socio-ecological framework. Filling these gaps is critical to inform precise, early-life interventions that can shape lifelong dietary habits and reduce the future burden of metabolic diseases.

To address these gaps, we conducted a large multicenter cross-sectional study across the seven major geographic regions of China [27], using a validated 7-day fluid intake questionnaire [26,28]. Our objective was to identify multilevel correlates of beverage consumption among Chinese young adults within a socio-ecological framework. By linking individual, behavioral, and environmental determinants, our findings aim at providing evidence to guide multilevel interventions, from nutrition education to policy measures, that promote healthier beverage choices and support national and global efforts to curb the growing metabolic disease burden.

## 2. Materials and Methods

### 2.1. Study Design

This multicenter, cross-sectional investigation was carried out from May to June 2023, targeting university students aged 18–25 years from the seven major geographic regions of China [27]. To achieve broad national coverage, a multistage stratified sampling approach was employed across the seven major geographic regions of China [27]. Site selection involved identifying representative institutions in each region: Tianjin Medical University (North), Jilin University (Northeast), Shanghai Jiao Tong University (East), Hainan Medical University (South), Yunnan Agricultural University (Southwest), Lanzhou University (Northwest), and Central South University (Central). These choices aimed at reflecting variations in climate, and sociocultural contexts. The study was guided by the socio-ecological framework, which posits that beverage consumption is shaped by individual (e.g., age, sex, and psychological status), interpersonal (e.g., family and social norms), and environmental (e.g., regional availability and socioeconomic conditions) factors.

The sample size was determined using the formula for cross-sectional studies:

(1) $$n=\frac{Z^{2}\times p\times \left(1-p\right)}{E^{2}}$$

where *Z* = 1.96 (for 95% confidence), *p* = 0.28 (based on prior surveys indicating that 28% of adults meeting adequate plain water intake), and *E* = 0.05 (margin of error) [29]. This yielded a base sample of approximately 313 per region. Adjusting for a 20% attrition rate increased the target to 322 per region, resulting in a total minimum of 2739 participants. Actual enrollment varied by regional recruitment success.

Ethical approval was granted by the Peking University Ethical Review Committee (IRB00001052-22119), with adherence to the Declaration of Helsinki principles. The study was registered in the Chinese Clinical Trial Registry (ChiCTR2400082286). All participants provided written informed consent prior to involvement.

### 2.2. Participants and Recruitment

Inclusion criteria focused on healthy undergraduate and graduate students aged 18–25 years. Exclusions encompassed current smokers, those with alcohol intake exceeding 20 g/day, individuals engaging in over 10 h of high-intensity exercise weekly, recent users of medications or supplements, and those with chronic conditions like gastrointestinal, kidney, metabolic, or cognitive issues.

Enrollment combined digital and physical methods to minimize bias. Online efforts used platforms such as WeChat and QQ for invitations, while campus-based tactics included posters, setup booths, and brief classroom talks. Materials outlined study objectives, processes, and criteria neutrally to encourage diverse participation.

### 2.3. Measurements

All data collection adhered to standardized protocols to maintain uniformity across participating sites. Demographic and lifestyle details were gathered through questionnaires administered under interviewer supervision to reduce incomplete entries and inaccuracies in reporting. Recruitment documents presented the study’s goals and methods objectively to mitigate participant self-selection biases.

#### 2.3.1. Beverage Intake Assessment

Daily fluids intake (DFI) was assessed using the validated 7-day, 24 h fluid intake diary (Liq.In7) [26,28], in which participants recorded all fluid consumption by type, volume, and drinking occasion. DFI was defined as the sum of all these categories. Beverage categories were defined according to the Chinese Beverage Classification Guideline (GB/T 10789-2015), [30] adapted to align with the Chinese Dietary Guidelines [31] and previous epidemiological studies [29]: plain water, sugar-sweetened beverages (SSBs), artificially sweetened beverages (ASBs), 100% fruit juices, coffee and tea, alcoholic beverages, and other beverages (Supplementary Table S1). Milk and milk derivatives were classified as food water according to the Dietary Guidelines and were excluded from beverage analyses [31], ensuring consistency with national dietary classification standards. To confirm appropriate classification, several experts in nutrition and dietary assessment were consulted, who agreed with this approach.

#### 2.3.2. Demographic and Socioeconomic Characteristics

Participants self-reported their age, gender, and ethnic group. Socioeconomic position was proxied using the Chinese City Tier System [32], which categorizes urban centers into first-tier, emerging first-tier, second-tier, and third-tier levels based on indicators of economic vitality, transit systems, built environment, and cultural prominence (as per data from the National Bureau of Statistics of China) [33].

#### 2.3.3. Lifestyle and Psychological Variables

Physical activity was measured using the validated International Physical Activity Questionnaire (IPAQ) [34]. Sleep quality was assessed with the Pittsburgh Sleep Quality Index (PSQI) [35]. Psychological status was evaluated using the Self-Rating Anxiety Scale (SAS) [36], and Self-Rating Depression Scale (SDS) [37], both validated for use in Chinese populations.

#### 2.3.4. Environmental Measures

Environmental exposures were characterized by site-specific mean daily temperature and relative humidity, recorded three times per day (10:00, 14:00, and 20:00) with portable sensors (Testo 608-H1; Testo SE, Germany; accuracy ±1 °C, ±0.1% RH) during the study period.

### 2.4. Quality Control

A comprehensive quality-assurance framework was established to maintain procedural consistency and safeguard the integrity of collected data. Study protocols and measurement instruments were derived from systematic literature review and consolidated expert input, with prior research demonstrating their reliability. A detailed operations manual outlined all field procedures, and staff underwent centralized training followed by competency certification before initiating fieldwork. Trained investigators, using regularly calibrated equipment, conducted all anthropometric measurements and biological sample collection under direct supervision, minimizing reliance on self-reported information.

To enhance the accuracy of fluid intake assessment, participants maintained a validated seven-day fluid diary with a 5 mL volume resolution. Nutrient content from foods was measured using the duplicate-portion method in accordance with national guidelines. Collected specimens were frozen at −20 °C, transported under cold-chain conditions, and analyzed in a central laboratory to reduce inter-laboratory variability.

Data management employed a double-entry system with independent cross-verification and automated logic checks to identify missing or inconsistent entries, which were promptly resolved. Personal identifiers were removed and replaced with anonymized codes, and electronic records were stored on encrypted servers with role-based access control. On-site quality control teams conducted routine monitoring visits, documenting and correcting any protocol deviations immediately.

### 2.5. Statistical Analysis

Descriptive statistics were first applied to characterize the study population and summarize beverage consumption patterns across regions and subgroups. The distribution of continuous variables was assessed using the Kolmogorov–Smirnov test, and because most variables were non-normally distributed, results are reported as medians with interquartile ranges (IQRs). Group differences were examined using the Mann–Whitney U test for two-level comparisons and the Kruskal–Wallis test for multiple group comparisons, with Dunn’s post hoc procedure applied for pairwise contrasts when overall significance was observed. Categorical variables were expressed as frequencies and percentages and compared using the χ^2^ test or Fisher’s exact test, as appropriate.

To explore determinants of beverage consumption, we constructed multivariable linear regression models for each beverage category (plain water, SSBs, ASBs, 100% fruit juices, coffee/tea, alcoholic beverages, and others), using daily intake volume as the dependent variable. All models were adjusted for covariates selected a priori based on theoretical and empirical relevance, including demographic and socioeconomic factors (age, sex, ethnicity, city-tier classification, and geographic region), lifestyle variables (physical activity from IPAQ and sleep quality from PSQI), and psychological status (SAS and SDS scores). Regression results are reported as regression coefficients (β) with corresponding 95% confidence intervals (CIs) and *p*-values.

Sex- and age-specific subgroup analyses were performed to evaluate whether beverage consumption patterns and their determinants varied by sex (male vs. female) or age group (18–19, 20–21, and 22–25 years). Age groups (18–19, 20–21, and 22–25 years) were defined based on the distribution of participants’ ages in the study population. Fully adjusted regression models were re-estimated within each subgroup. To formally examine effect modification, interaction terms between beverage categories and sex or age group were included in the multivariable models, and the statistical significance of the interaction terms was assessed.

All analyses were performed using R software (version 4.3.2; R Foundation for Statistical Computing, Vienna, Austria), and statistical significance was defined as two-sided *p* < 0.05. *p*-values between 0.05 and 0.10 were interpreted as borderline significant.

## 3. Results

### 3.1. Characteristics of Participants

**Figure 1:**
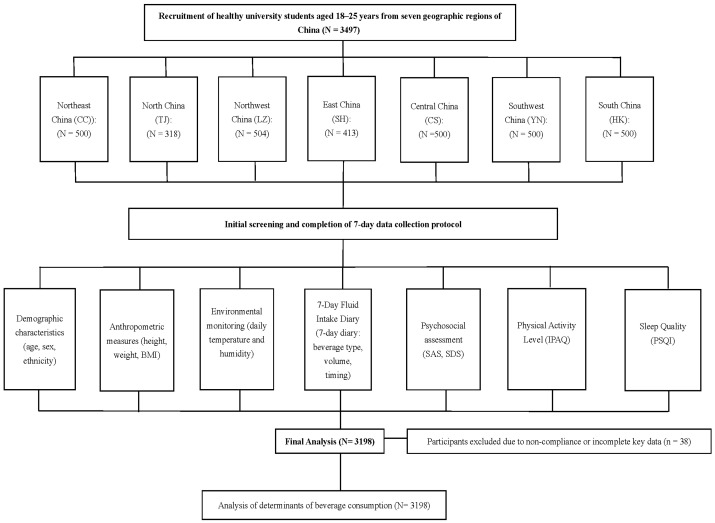
Flowchart of participant recruitment, data collection, and analysis. This flowchart illustrates participant recruitment, data collection, and inclusion in the final analysis. A total of 3497 healthy university students aged 18–25 years were recruited from the seven major geographic regions of China. Following initial screening, participants completed a 7-day protocol including demographic and anthropometric assessments, 7-day fluid intake diaries, physical activity evaluation (International Physical Activity Questionnaire, IPAQ), sleep quality assessment (Pittsburgh Sleep Quality Index, PSQI), psychosocial measures (Self-Rating Anxiety Scale, SAS; Self-Rating Depression Scale, SDS), and environmental monitoring. After excluding 38 participants for non-compliance or incomplete data, 3198 participants were included in the final analysis of determinants of beverage consumptions.

The recruitment flowchart is shown in Figure 1, and the participants’ characteristics are summarized in Table 1. A total of 3198 healthy young adults were included in the final analysis (median age: 20.0 years; IQR: 19.0–20.0). Among them, 54.9% were male (*n* = 1748) and 45.1% were female (*n* = 1437). Most participants were of Han ethnicity (88.1%), with minorities accounting for 11.3%. Participants were recruited from the seven major geographic regions of China, each contributing 9.9–16.2% of the sample, ensuring balanced regional representation. According to BMI categories, 63.0% were normal weight, 16.4% overweight, 3.8% obese, and 15.9% underweight.

Regarding lifestyle, 17.3% reported low physical activity, while 41.3% and 40.5% reported moderate and high levels, respectively. Sleep quality was modest (median PSQI: 6.0, IQR: 4.0–7.0). Median daily energy intake was 1714.5 kcal with balanced macronutrient distribution. Psychological assessments (SAS: 35.0; SDS: 42.0) indicated generally normal mental health. Median daily fluid intake was 1259.9 mL, predominantly from plain water; intake of SSBs, ASBs, 100% fruit juice, and alcoholic beverages was low for most participants. In addition, the descriptive statistics of daily beverage intake (mL/day) by participant characteristics are summarized in Supplementary Table S2.

### 3.2. Overall Determinants of Beverage Consumption

As shown in Table 2, sex, age, and sleep quality were the strongest predictors of beverage consumption among young adults. Plain water intake increased with age (β = 33.42, 95% CI: 19.92–46.91, *p* < 0.001). Females consumed less plain water (β = −112.75, 95% CI: −147.98 to −77.53, *p* < 0.001), SSBs (β = −23.59, 95% CI: −34.60 to −12.57, *p* < 0.001), ASB (β = −4.01, 95% CI: −6.45 to −1.58, *p* = 0.001), and alcoholic beverages (β = –3.96, 95% CI: −6.18 to −1.74, *p* < 0.001) than males. Intake increased with age for plain water (β = 33.42, 95% CI: 19.92–46.91, *p* < 0.001), coffee and tea (β = 7.20, 95% CI: 3.83–10.58, *p* < 0.001), and alcoholic beverages (β = 1.76, 95% CI: 0.91–2.61, *p* < 0.001). Poorer sleep quality (higher PSQI scores) was associated with higher SSBs consumption (β = 2.55, 95% CI: 0.36–4.73, *p* = 0.023), whereas 100% fruit juice showed no significant predictors. Physical activity, ethnicity, regional temperature and humanity, and socioeconomic tier had minimal or inconsistent associations across beverages.

### 3.3. Sex-Stratified Analysis

Supplementary Table S3 demonstrates gender-specific associations between beverage intake and sociodemographic, behavioral, and environmental factors. Gender differences remained evident after adjustment, particularly for plain water and SSBs intake. Age remained positively associated with plain water intake in both sexes, but the effect was stronger in females (males: β = 23.75, 95% CI: 3.94–43.57, *p* = 0.019; females: β = 46.17, 95% CI: 27.74–64.60, *p* < 0.001). Coffee and tea intake increased with age in males (β = 9.39, 95% CI: 3.78–14.99, *p* = 0.001). Among females, non-Han participants consumed more SSBs than Han participants (β = 5.31, 95% CI: 1.22–9.39, *p* = 0.011), and higher SDS scores were associated with greater SSBs intake (β = 1.35, 95% CI: 0.16–2.54, *p* = 0.026).

### 3.4. Age-Stratified Analysis

Supplementary Table S4 presents age-specific associations between beverage intake and sociodemographic, behavioral, and environmental factors. Across age groups, variations in beverage consumption were primarily driven by gender and environmental conditions such as temperature and humidity. Among participants aged 18–21 years, females consumed less plain water (18–19 years: β = −116.62, 95% CI: –166.03 to −67.21, *p* < 0.001; 20–21 years: β = −125.11, 95% CI: –180.27 to –69.96, *p* < 0.001) and SSBs (18–19 years: β = −19.92, 95% CI: −35.85 to –3.99, *p* = 0.014; 20–21 years: β = −24.28, 95% CI: −40.69 to –7.88, *p* = 0.004), whereas no significant gender differences were observed among participants aged 22–25 years. Among participants aged 22–25 years, SSBs intake was positively associated with socioeconomic tier 1.5 (β = 109.11, 95% CI: 35.64–182.58, *p* = 0.004) and higher regional temperature (β = 31.34, 95% CI: 13.11–49.57, *p* = 0.001), and inversely associated with humidity (β = −5.35, 95% CI: −8.32 to −2.39, *p* < 0.001).

Coffee and tea intake increased with regional temperature and decreased with humidity in participants aged 22–25 years (temperature: β = 22.63, 95% CI: 3.58–41.68, *p* = 0.020; humidity: β = −3.57, 95% CI: −6.67 to −0.46, *p* = 0.024). Associations in younger age groups were not statistically significant.

### 3.5. Interaction Between Age Group and Gender

To further investigate potential age-by-sex interactions suggested by the findings found in previous gender- and age-stratified analysis, multivariable regression models were performed (Supplementary Table S5). Results confirmed that both age and gender jointly influenced beverage consumption, especially for plain water and SSBs intake. Plain water consumption was significantly lower in females aged 20–21 years (β = −111.05, 95% CI: −161.79 to −60.31, *p* < 0.001) and ≥22 years (β = −120.68, 95% CI: 29.737, 211.623, *p* = 0.009) compared to their male counterparts, and intake increased with age in both sexes. Intake of SSBs was also lower in females relative to males (β = −21.81, 95% CI: −37.66 to −5.95, *p* = 0.007), with higher PSQI scores associated with greater consumption. Coffee and tea consumption increased among participants aged ≥22 years (β = 29.71, 95% CI: 6.89–52.52, *p* = 0.011).

## 4. Discussion

### 4.1. Principal Findings

In this nationally and regionally representative study, sex, age, and sleep quality emerged as the primary determinants of beverage intake among young Chinese adults. Females consumed less plain water and SSBs than males, whereas intake of plain water, SSBs, and coffee and tea increased with age. Poorer sleep quality was linked to higher SSBs consumption. Environmental and socioeconomic factors also influenced SSBs intake in older participants.

These findings provide actionable insights for public health strategies aimed at promoting healthier beverage habits. Policies aligned with the Healthy China 2030 Action Plan, including sugar reduction initiatives and upstream measures such as SSB taxation, can be tailored to high-risk groups identified by demographic and behavioral characteristics. Targeted interventions addressing modifiable behaviors, such as sleep quality, could further foster healthier hydration and reduce long-term metabolic risk among young adults.

### 4.2. Comparison with Previous Studies and Possible Explanations

Our findings align with previous studies reporting lower consumption of plain water and SSBs among women [29], which is partially explained by physiological differences such as lower body water content and metabolic rates in females [31]. Additionally, females may consume fewer less SSBs due to greater health consciousness and body image concerns, leading to a preference for lower-calorie or unsweetened beverages options [38]. Interestingly, our sex-stratified analysis revealed that females with higher depressive symptoms (elevated SDS scores) were more likely to consume SSBs than males, suggesting a gender-specific response of beverage consumption to emotional stress [39].

Beyond sex differences, SSBs intake in the 22–25 age group was positively associated with socioeconomic tier 1.5, potentially reflecting greater exposure to rapid urbanization and economic development in emerging first-tier cities [3,8,40]. Higher regional temperatures were also linked to greater SSBs consumption, consistent with evidence that rising ambient temperatures increase demand for SSBs [19]. These findings collectively highlight the influence of urbanization and environmental stressors on beverage choices in later stages of early adulthood.

Importantly, behavioral and psychological factors also shaped SSBs intake. Poor sleep quality was positively associated with SSBs consumption, aligning with previous study that revealed short sleep duration potentially dysregulate appetite-related hormones and activate brain reward pathways, thereby increasing cravings for sweetened beverages [41,42,43]. These results address the need for integrated interventions that address both lifestyle behaviors and mental health to curb SSBs consumption in vulnerable subgroups.

Coffee and tea consumption increased with age, and warmer, drier conditions were associated with higher intake in the 22–25-year age group. These findings are consistent with data from the China Health and Nutrition Survey [44] and may reflect greater health awareness, cultural preferences for caffeinated beverages as social or productivity aids, and adaptation to adult routines among older young adults [45].

### 4.3. Strengths, Limitations, and Future Directions

There are several strengths. This study is the first nationally and regionally representative multicenter investigation to systematically examine determinants of beverage consumption in healthy young Chinese adults. We comprehensively assessed demographic, environmental, psychological, and behavioral factors using validated questionnaires and standardized protocols across seven study sites, ensuring methodological consistency. Beverage intake was measured with high precision through a validated seven-day fluid intake questionnaire and calibrated measuring bottles, thereby minimizing recall bias. This rigorous design strengthens the reliability and generalizability of our findings and facilitates the identification of subgroups for targeted public health interventions.

However, several limitations must be acknowledged. The cross-sectional design precludes causal inference, and findings are specific to university students aged 18–25 years, limiting generalizability to other young adult and age populations. Data were collected from May to July to minimize seasonal variation. Future studies employing longitudinal or interventional designs are needed to establish causality and to further investigate additional determinants, including genetic predisposition, metabolic markers, and psychological traits, which may explain individual variability. Incorporating objective biomarkers of hydration and sugar intake would provide mechanistic insights. The high-risk subgroups identified in this study should be prioritized in intervention research to promote healthier beverage habits. Future studies also should assess beverage consumption across multiple seasons to capture seasonal variability and improve generalizability.

### 4.4. Public Health Implications

Our findings underscore the urgent need to integrate behavioral, environmental, and policy-level strategies to improve beverage choices among young adults. Current Chinese initiatives, such as the *Healthy China 2030 Action Plan* [25], focus primarily on individual lifestyle modification. International experience demonstrates the potential effectiveness of fiscal and educational measures. SSB taxation in Mexico and the UK has reduced purchases by 10–20% within two years [20,21]. Most countries tax based on sugar content and/or target only SSBs, while 100% fruit juices are generally exempt and low-calorie sweetened (LCS) drinks are rarely included. Evaluations of the Mexican soda tax indicate partial substitution toward bottled water, without significant increases in fruit juice or LCS drinks. School-based policies often exclude LCS drinks but allow controlled portions of 100% fruit juice, highlighting the need to consider substitution and caloric equivalence. Public education campaigns promoting low-calorie alternatives, such as water or LCS drinks, further support these measures, as seen in New York City, the UK, and Tonga [14].

The World Health Organization (WHO) recommends population-wide strategies to reduce free sugar intake to <10% of total energy consumption, with further benefits at <5% [24], providing an evidence-based target for policy design. For China, a combined strategy targeting young adults—including sugar-content-based taxes, school-based healthy beverage standards, public awareness campaigns, and improved access to safe drinking water—could provide a feasible, multilevel approach. Such interventions may synergistically reduce sugar consumption when integrated with measures to restrict marketing of SSBs to youth and mandatory front-of-pack labeling [14].

## 5. Conclusions

In conclusion, sex, age, and sleep quality were the primary determinants of beverage consumption in young Chinese adults, with clear age- and sex-specific patterns. Early adulthood appears to be a critical window for establishing lifelong hydration habits, making this population an ideal target for public health interventions. Combining behavior-change programs with structural policies, such as SSB taxation, marketing regulation, and infrastructure improvements, could help reduce the burden of obesity, T2DM, and other NCDs. Future research should use longitudinal and interventional designs to confirm causality, explore mechanisms and evaluate the real-world impact of policy measures aligned with both *Healthy China 2030* and WHO NCD Global Action Plan targets.

## Figures and Tables

**Table 1:** Characteristics of participants.

Variable	Overall (*N* = 3198)
Demographic characteristics	
Age (years)	20.000 (19.000, 20.000)
Ethnicity, *n* (%)	
Han	2817 (88.1%)
Minority	361 (11.3%)
Sex, *n* (%)	
Female	1437 (45.1%)
Male	1748 (54.9%)
BMI category, *n* (%)	
Underweight	509 (15.9%)
Normal weight	2016 (63.0%)
Overweight	526 (16.4%)
Obesity	122 (3.8%)
Region, *n* (%)	
Northeast China (Changchun, CC)	499 (15.6%)
North China (Tianjin, TJ)	316 (9.9%)
Northwest China (Lanzhou, LZ)	502 (15.7%)
East China (Shanghai, SH)	398 (12.4%)
Central China (Changsha, CS)	498 (15.6%)
Southwest China (Yunnan, YN)	466 (14.6%)
South China (Haikou, HK)	519 (16.2%)
Lifestyle factors	
Physical activity level, *n* (%)	
Low	552 (17.3%)
Medium	1322 (41.3%)
High	1294 (40.5%)
PSQI score	6.000 (4.000, 7.000)
Psychological factors	
SAS score	35.000 (32.000, 39.000)
SDS score	42.000 (38.000, 46.000)
Beverage intake variables	
DFI (mL/day)	1259.910 (938.888, 1593.000)
Plain Water (mL/day)	1259.910 (938.888, 1593.000)
Coffee Tea (mL/day)	1050.000 (750.000, 1356.750)
SSBs (mL/day)	0.000 (0.000, 50.000)
100% Fruit Juices (mL/day)	50.000 (0.000, 150.000)
Other Beverages (mL/day)	0.000 (0.000, 50.000)

Data are presented as median (interquartile range, IQR) for continuous variables and *n* (%) for categorical variables. Psychological status was assessed using the Self-Rating Anxiety Scale (SAS) and Self-Rating Depression Scale (SDS). Beverage intake variables represent median daily consumption in mL/day. Abbreviations: SSBs = sugar-sweetened beverages; DB = dairy beverages; WB = water-based beverages, etc. ASBs and alcoholic beverages were excluded from the table because their median intake was 0 in all regions. Region codes: CC = Changchun (Northeast); TJ = Tianjin (North); LZ = Lanzhou (Northwest); SH = Shanghai (East); CS = Changsha (Central); YN = Yunnan (Southwest); HK = Haikou (South).

**Table 2:** Multivariable linear regression of factors associated with beverage consumption in adults.

Beverage Types	Variables	β (95% CI)	*p*
Plain Water (mL/day)	Gender (Female vs. Male)	−112.754 [−147.976, −77.533]	<0.001
	Age(years)	33.415 [19.92, 46.909]	<0.001
	Ethnicity (non-Han)	−40.664 [−95.027, 13.698]	0.143
	PA Level (Moderate)	−10.179 [−59.877, 39.519]	0.688
	PA Level (High)	25.517 [−24.851, 75.886]	0.321
	PSQI Score	−5.085 [−12.093, 1.923]	0.155
	SAS Score	−1.742 [−4.867, 1.383]	0.274
	SDS Score	2.115 [−0.924, 5.154]	0.173
	Region Temperature (°C)	32.064 [−56.828, 120.956]	0.259
	Region Temperature (%)	−1.832 [−17.596, 13.933]	0.663
	Socioeconomical Tier (1.5)	191.916 [−300.848, 684.68]	0.235
	Socioeconomical Tier (2)	359.368 [−177.328, 896.065]	0.102
Coffee and Tea (mL/day)	Gender (Female vs. Male)	5.195 [−3.629, 14.019]	0.248
	Age(years)	7.202 [3.829, 10.575]	<0.001
	Ethnicity (non-Han)	3.554 [−10.06, 17.168]	0.609
	PA Level (Moderate)	−5.095 [−17.54, 7.349]	0.422
	PA Level (High)	3.358 [−9.262, 15.979]	0.602
	PSQI Score	1.379 [−0.352, 3.111]	0.118
	SAS Score	0.817 [0.035, 1.6]	0.041
	SDS Score	−0.464 [−1.224, 0.296]	0.232
	Region Temperature (°C)	8.715 [−1.303, 18.733]	0.065
	Region Temperature (%)	−1.371 [−3.167, 0.425]	0.081
	Socioeconomical Tier (1.5)	15.23 [−40.036, 70.495]	0.361
	Socioeconomical Tier (2)	9.495 [−51.335, 70.325]	0.569
100% Fruit Juices (mL/day)	Gender (Female vs. Male)	4.665 [−0.214, 9.544]	0.061
	Age(years)	0.292 [−1.578, 2.161]	0.76
	Ethnicity (non-Han)	3.824 [−3.707, 11.355]	0.319
	PA Level (Moderate)	−3.012 [−9.897, 3.873]	0.391
	PA Level (High)	−3.365 [−10.342, 3.613]	0.344
	PSQI Score	0.171 [−0.801, 1.143]	0.73
	SAS Score	0.369 [−0.064, 0.802]	0.095
	SDS Score	−0.027 [−0.448, 0.395]	0.902
	Region Temperature (°C)	−9.54 [−25.794, 6.713]	0.128
	Region Temperature (%)	1.904 [−0.975, 4.782]	0.105
	Socioeconomical Tier (1.5)	−28.878 [−119.073, 61.317]	0.304
	Socioeconomical Tier (2)	−24.519 [−122.579, 73.541]	0.396
SSBs (mL/day)	Gender (Female vs. Male)	−23.588 [−34.603, −12.573]	<0.001
	Age(years)	0.259 [−3.96, 4.479]	0.904
	Ethnicity (non-Han)	−11.785 [−28.785, 5.215]	0.174
	PA Level (Moderate)	−5.125 [−20.666, 10.416]	0.518
	PA Level (High)	−0.934 [−16.686, 14.818]	0.907
	PSQI Score	2.545 [0.355, 4.734]	0.023
	SAS Score	−0.383 [−1.36, 0.594]	0.443
	SDS Score	0.722 [−0.228, 1.673]	0.136
	Region Temperature (°C)	12.507 [−11.644, 36.657]	0.157
	Region Temperature (%)	−2.038 [−6.323, 2.248]	0.178
Alcoholic Beverages (mL/day)	Gender (Female vs. Male)	−3.96 [−6.175, −1.744]	<0.001
	Age(years)	1.761 [0.913, 2.608]	<0.001
	Ethnicity (non-Han)	4.022 [0.603, 7.44]	0.021
	PA Level (Moderate)	−0.918 [−4.043, 2.207]	0.565
	PA Level (High)	−0.077 [−3.246, 3.092]	0.962
	PSQI Score	−0.109 [−0.546, 0.327]	0.623
	SAS Score	−0.16 [−0.357, 0.037]	0.111
	SDS Score	0.15 [−0.041, 0.341]	0.124
	Region Temperature (°C)	0.815 [−2.116, 3.746]	0.358
	Region Temperature (%)	−0.125 [−0.648, 0.398]	0.413
	Socioeconomical Tier (1.5)	1.068 [−15.118, 17.254]	0.807
	Socioeconomical Tier (2)	0.217 [−17.541, 17.975]	0.963
Other Beverages (mL/day)	Gender (Female vs. Male)	−2.822 [−7.241, 1.598]	0.211
	Age(years)	0.447 [−1.247, 2.141]	0.605
	Ethnicity (non-Han)	−2.222 [−9.044, 4.6]	0.523
	PA Level (Moderate)	4.88 [−1.356, 11.117]	0.125
	PA Level (High)	7.973 [1.653, 14.294]	0.013
	PSQI Score	−0.109 [−0.991, 0.772]	0.808
	SAS Score	0.084 [−0.308, 0.476]	0.673
	SDS Score	−0.086 [−0.468, 0.295]	0.658
	Region Temperature (°C)	−5.805 [−24.77, 13.16]	0.318
	Region Temperature (%)	0.607 [−2.75, 3.964]	0.517
	Socioeconomical Tier (1.5)	−21.089 [−126.385, 84.206]	0.479
	Socioeconomical Tier (2)	−31.158 [−145.538, 83.222]	0.361

Values are presented as β coefficients with 95% confidence intervals (CIs) and corresponding *p*-values derived from multivariable linear regression models. Models were adjusted for gender, age, ethnicity, physical activity (PA) level, Pittsburgh Sleep Quality Index (PSQI) score, Self-rating Anxiety Scale (SAS) score, Self-rating Depression Scale (SDS) score, regional temperature, and socioeconomic tier. Statistical significance is denoted at *p* < 0.05. Abbreviations: SSBs, sugar-sweetened beverages; ASBs, artificially sweetened beverages.

## Data Availability

The original contributions presented in the study are included in the article/Supplementary Material, further inquiries can be directed to the corresponding authors.

## Supplementary Materials

The following supporting information can be downloaded at: https://www.mdpi.com/article/10.3390/foods14213687/s1, Supplementary Table S1: Classification of Beverage Types. Beverage classification was based on the Chinese Beverage Classification Guideline (GB/T 10789-2015), adapted to align with the Chinese Dietary Guidelines and previous epidemiological studies. Seven categories were analyzed: plain water, sugar-sweetened beverages (SSBs), artificially sweetened beverages (ASBs), 100% fruit juices, coffee and tea, alcoholic beverages, and other beverages. Milk and milk derivatives were classified as food water according to the Chinese Dietary Guidelines and were excluded from beverage analyses. Supplementary Table S2: Descriptive Statistics of Daily Beverage Intake (mL/day) Stratified by Participant Characteristics. This table presents descriptive statistics for daily beverage intake, stratified by various participant characteristics, including gender, age group, ethnicity, physical activity level, and city tier. The values are reported as medians with interquartile ranges (IQR) in parentheses, except for categorical variables like fruit juices and alcohol, where intake was reported as zero for a large portion of the sample. Supplementary Table S3: Multivariable Linear Regression of Factors Associated with Beverage Consumption Subgroup Analysis by Sex. Values are presented as β (95% CI) with corresponding P-values from multivariable linear regression models. Beverage categories included plain water, coffee/tea, fruit juices, sugar-sweetened beverages (SSBs), artificially sweetened beverages (ASB), alcoholic beverages, and other beverages. Independent variables included age (continuous), ethnicity (non-Han vs. Han), physical activity (PA) level (moderate and high, reference = low), sleep quality score (PSQI_S), anxiety score (SAS), depression score (SDS), regional average temperature (°C) and humidity (%), and socioeconomic tier (1.5 and 2, reference = 1). All models were adjusted for the above covariates to minimize confounding. Statistical significance is denoted at *p* < 0.05. Supplementary Table S4: Multivariable Linear Regression of Factors Associated with Beverage Consumption Subgroup Analysis by Age Group. Values are presented as β (95% CI) with corresponding *p*-values from multivariable linear regression models. Beverage categories include plain water, coffee/tea, fruit juices, sugar-sweetened beverages (SSBs), artificially sweetened beverages (ASB), alcoholic beverages, and other beverages. Covariates include gender (female vs. male), ethnicity (non-Han vs. Han), physical activity (PA) level (moderate or high vs. low), sleep quality (PSQI score), anxiety (SAS score), depression (SDS score), regional temperature (°C), regional humidity (%), and socioeconomic tier (1.5 and 2 vs. 1). All models were mutually adjusted. Statistical significance is denoted at *p* < 0.05. Supplementary Table S5: Interaction Effects of Age, Sex, and Other Covariates on Beverage Consumption. This table presents the beta coefficients (β) with 95% confidence intervals (CI) and *p*-values from a multivariable linear regression model examining factors associated with daily beverage intake (in milliliters or equivalent units, assumed based on context). The model includes main effects for age group (reference: 18-19 years), gender (reference: male), ethnicity (reference: Han), physical activity (PA) level (reference: low), Pittsburgh Sleep Quality Index (PSQI) score, Self-Rating Anxiety Scale (SAS) score, Self-Rating Depression Scale (SDS) score, regional average temperature (°C), regional average humidity (%), and Socioeconomic tier (reference: tier 1 = first-tier cities; tier 1.5 = emerging first-tier cities; tier 2 = second-tier cities).Interaction terms are included for age group by gender (e.g., Age group (20-21 years): Female). Beverage types analyzed include Plain Water, Coffee and Tea, Fruit Juices, Sugar-Sweetened Beverages (SSBs), Artificially Sweetened Beverages (ASB), Alcoholic Beverages, and Other Beverages. Positive β values indicate higher intake associated with the variable, while negative values indicate lower intake. Statistical significance is denoted at *p* < 0.05. All analyses adjust for potential confounders listed. Supplementary Figure S1: Mean beverage intake (DFI) composition (mL/day) by gender (including overall). The chart displays the mean daily intake of various beverage categories for the overall population, males, and females. The total height of each bar represents the mean total DFI, and the colored segments represent the mean intake (mL/day) of each specific beverage category. Abbreviations: DFI, Drinking Fluid Intake; SSBs, Sugar-Sweetened Beverages; ASB, Artificially Sweetened Beverages. Supplementary Figure S2: Mean beverage intake (DFI) composition (mL/day) by region (including overall). The chart displays the mean daily intake of various beverage categories for the overall population and across seven different regions (CC, TJ, LZ, SH, CS, YN, HK). The total height of each bar represents the mean total DFI, and the colored segments represent the mean intake (mL/day) of each specific beverage category. Abbreviations: DFI, Drinking Fluid Intake; SSBs, Sugar-Sweetened Beverages; ASB, Artificially Sweetened Beverages.

## Abbreviations

The following abbreviations are used in this manuscript:

T2DM	Type 2 Diabetes Mellitus
CVD	Cardiovascular Disease
SSBs	Sugar-Sweetened Beverages
ASB	Artificially Sweetened Beverage
DFI	Daily Fluids Intake
CHNS	China Health and Nutrition Survey
CARDIA	Coronary Artery Risk Development in Young Adults
WHO	World Health Organization
GB/T	Guobiao Standard (National Standard of China)
IRB	Institutional Review Board
ChiCTR	Chinese Clinical Trial Registry
IPAQ	International Physical Activity Questionnaire
PSQI	Pittsburgh Sleep Quality Index
SAS	Self-Rating Anxiety Scale
SDS	Self-Rating Depression Scale
IQR	Interquartile Range
CI	Confidence Interval

## References

[1] Global Burden of Disease Collaborative Network Global Burden of Disease (GBD) 2000–2019. https://vizhub.healthdata.org/gbd-results/.

[2] The International Diabetes Federation IDF Atlas Reports: Diabetes and Kidney Disease. https://diabetesatlas.org/resources/idf-diabetes-atlas-reports/diabetes-and-kidney-disease/.

[3] Pan X.F., Wang L., Pan A. (2021). Epidemiology and determinants of obesity in China. Lancet Diabetes Endocrinol..

[4] Chen Y., Peng Q., Yang Y., Zheng S., Wang Y., Lu W. (2019). The prevalence and increasing trends of overweight, general obesity, and abdominal obesity among Chinese adults: A repeated cross-sectional study. BMC Public Health.

[5] Leunissen R.W., Kerkhof G.F., Stijnen T., Hokken-Koelega A. (2009). Timing and tempo of first-year rapid growth in relation to cardiovascular and metabolic risk profile in early adulthood. JAMA.

[6] Scott J., Agarwala A., Baker-Smith C.M., Feinstein M.J., Jakubowski K., Kaar J., Parekh N., Patel K.V., Stephens J. (2025). Cardiovascular Health in the Transition From Adolescence to Emerging Adulthood: A Scientific Statement From the American Heart Association. J. Am. Heart Assoc..

[7] Sun J., Qiao Y., Zhao M., Magnussen C.G., Xi B. (2023). Global, regional, and national burden of cardiovascular diseases in youths and young adults aged 15–39 years in 204 countries/territories, 1990–2019: A systematic analysis of Global Burden of Disease Study 2019. BMC Med..

[8] Pan F.L.D., Zhang T.W., Mao W.F., Liang D., Liu A.D., Li J.W. (2022). Consumption status of sugar-sweetened beverages and free sugar intake among urban residents aged 3 years and above in China. Chin. J. Food Hyg..

[9] Jakob J., Stalder O., Kali T., Pruvot E., Pletcher M.J., Rana J.S., Sidney S., Auer R. (2022). The Coronary Artery Risk Development in Young Adults (CARDIA) Study. Am. J. Med..

[10] de Oliveira Otto M.C., Anderson C.A.M., Dearborn J.L., Ferranti E.P., Mozaffarian D., Rao G., Wylie-Rosett J., Lichtenstein A.H. (2018). Dietary Diversity: Implications for Obesity Prevention in Adult Populations: A Science Advisory From the American Heart Association. Circulation.

[11] Ma L., Hu Y., Alperet D.J., Liu G., Malik V., Manson J.E., Rimm E.B., Hu F.B., Sun Q. (2023). Beverage consumption and mortality among adults with type 2 diabetes: Prospective cohort study. BMJ.

[12] Di Maso M., Boffetta P., Negri E., La Vecchia C., Bravi F. (2021). Caffeinated Coffee Consumption and Health Outcomes in the US Population: A Dose-Response Meta-Analysis and Estimation of Disease Cases and Deaths Avoided. Adv. Nutr..

[13] Salgado M.V., Penko J., Fernandez A., Konfino J., Coxson P.G., Bibbins-Domingo K., Mejia R. (2020). Projected impact of a reduction in sugar-sweetened beverage consumption on diabetes and cardiovascular disease in Argentina: A modeling study. PLoS Med..

[14] Popkin B.M., Hawkes C. (2016). Sweetening of the global diet, particularly beverages: Patterns, trends, and policy responses. Lancet Diabetes Endocrinol..

[15] Chen I.J., Liu C.Y., Chiu J.P., Hsu C.H. (2016). Therapeutic effect of high-dose green tea extract on weight reduction: A randomized, double-blind, placebo-controlled clinical trial. Clin. Nutr..

[16] Bag S., Mondal A., Majumder A., Banik A. (2022). Tea and its phytochemicals: Hidden health benefits & modulation of signaling cascade by phytochemicals. Food Chem..

[17] Guo H.A.-O., Phung D.A.-O., Chu C. (2021). Sociodemographic, lifestyle, behavioral, and parental factors associated with sugar-sweetened beverage consumption in children in China. PLoS ONE.

[18] Paes V.M., Hesketh K., O’Malley C., Moore H., Summerbell C., Griffin S. (2015). Obes Rev.: Determinants of sugar-sweetened beverage consumption in young children: A systematic review. J. Aust. Tradit.-Med. Soc..

[19] He P., Xu Z., Chan D., Liu P., Bai Y. (2025). Rising temperatures increase added sugar intake disproportionately in disadvantaged groups in the USA. Nat. Clim. Change.

[20] Colchero M.A., Rivera-Dommarco J., Popkin B.M., Ng S.W. (2017). In Mexico, Evidence Of Sustained Consumer Response Two Years After Implementing A Sugar-Sweetened Beverage Tax. Health Aff..

[21] World Bank Mexico: Population, Total 2017. https://data.worldbank.org/indicator/SP.POP.TOTL?locations=MX.

[22] Scarborough P., Adhikari V., Harrington R.A., Elhussein A., Briggs A., Rayner M., Adams J., Cummins S., Penney T., White M. (2020). Impact of the announcement and implementation of the UK Soft Drinks Industry Levy on sugar content, price, product size and number of available soft drinks in the UK, 2015–2019: A controlled interrupted time series analysis. PLoS Med..

[23] World Health Organization Noncommunicable Diseases. https://www.who.int/news-room/fact-sheets/detail/noncommunicable-diseases.

[24] WHO (2015). Guidelines Approved by the Guidelines Review Committee. Guideline: Sugars Intake for Adults and Children.

[25] Council C.S. National Nutrition Plan (2017–2030). https://leap.unep.org/en/countries/cn/national-legislation/national-nutrition-plan-2017-2030.

[26] Zhang N.Z.J., Ma G.S. (2024). Development, validation, and application of the 7-day 24-hour drinking water survey questionnaire. Chin. Food Nutr..

[27] Toponym Group of National Topographical Bureau (1983). Toponym Collections of China-Toponym Indexes of China.

[28] Zhang J.F.Z.N., He H.R., Ma G.S. (2025). Reliability and validity of the 7-day 24-hour drinking water survey questionnaire. Chin. Food Nutr..

[29] Zhang N., Morin C., Guelinckx I., Moreno L.A., Kavouras S.A., Gandy J., Martinez H., Salas-Salvadó J., Ma G. (2018). Fluid intake in urban China: Results of the 2016 Liq.In (7) national cross-sectional surveys. Eur. J. Nutr..

[30] (2015). General Rules for Beverage.

[31] Chinese Nutrition Society (2022). Chinese Dietary Guidelines (2022).

[32] National Bureau of Statistics of China China Statistical Yearbook. http://www.stats.gov.cn/sj/ndsj/2024/indexeh.htm.

[33] Huang Y., Wei L., Liu G., Cui W., Xie F., Deng X. (2023). “Inspiring” Policy Transfer: Analysis of Urban Renewal in Four First-Tier Chinese Cities. Land.

[34] Ekingen T., Sob C., Hartmann C., Rühli F.J., Matthes K.L., Staub K., Bender N. (2022). Associations between hydration status, body composition, sociodemographic and lifestyle factors in the general population: A cross-sectional study. BMC Public Health.

[35] Lee K.W., Shin D., Song W.O. (2016). Total Water Intake from Beverages and Foods Is Associated with Energy Intake and Eating Behaviors in Korean Adults. Nutrients.

[36] Zung W.W. (1971). A rating instrument for anxiety disorders. Psychosomatics.

[37] Zung W.W. (1965). A self-rating depression scale. Arch. Gen. Psychiatry.

[38] Gui Z.-H., Zhu Y.-N., Cai L., Sun F.-H., Ma Y.-H., Jing J., Chen Y.-J. (2017). Sugar-sweetened beverage consumption and risks of obesity and hypertension in Chinese children and adolescents: A national cross-sectional analysis. Nutrients.

[39] Gillespie K.M., Kemps E., White M.J., Bartlett S.E. (2025). The association of dietary components with depression and anxiety symptoms: Findings from a cross-sectional survey. Front. Nutr..

[40] Xu Y., Lu J., Li M., Wang T., Wang K., Cao Q., Ding Y., Xiang Y., Wang S., Yang Q. (2024). Diabetes in China part 1: Epidemiology and risk factors. Lancet Public Health.

[41] St-Onge M.P., McReynolds A., Trivedi Z.B., Roberts A.L., Sy M., Hirsch J. (2012). Sleep restriction leads to increased activation of brain regions sensitive to food stimuli. Am. J. Clin. Nutr..

[42] Spiegel K., Tasali E., Penev P., Van Cauter E. (2004). Brief communication: Sleep curtailment in healthy young men is associated with decreased leptin levels, elevated ghrelin levels, and increased hunger and appetite. Ann. Intern. Med..

[43] Dashti H.S., Scheer F.A., Jacques P.F., Lamon-Fava S., Ordovás J.M. (2015). Short sleep duration and dietary intake: Epidemiologic evidence, mechanisms, and health implications. Adv. Nutr..

[44] Mitchell D.C., Knight C.A., Hockenberry J., Teplansky R., Hartman T.J. (2014). Beverage caffeine intakes in the U.S. Food Chem. Toxicol..

[45] Landais E., Moskal A., Mullee A., Nicolas G., Gunter M.J., Huybrechts I., Overvad K., Roswall N., Affret A., Fagherazzi G. (2018). Coffee and Tea Consumption and the Contribution of Their Added Ingredients to Total Energy and Nutrient Intakes in 10 European Countries: Benchmark Data from the Late 1990s. Nutrients.

