# Supplementary material for: Determinants of Beverage Consumption in Young Adults: A Multicenter Cross-Sectional Study Across Seven Major Geographic Regions of China
Source: Foods. 2025 Oct 29;14(21):3687. doi: 10.3390/foods14213687 (PMC12607530; doi:10.3390/foods14213687)
Supplement: Supplementary file 1 [file foods-14-03687-s001.zip › foods-3930564-supplementary.pdf]

Supplementary Materials

Content

Supplementary Tables 2
Supplementary Table S1 Classification of Beverage Types 2
Supplementary Table S2. Descriptive Statistics of Daily Beverage Intake (mL/day) Stratified by Participant Characteristics 3
Supplementary Table S3 Multivariable Linear Regression of Factors Associated with Beverage Consumption Subgroup Analysis by Sex........ 5
Supplementary Table S4. Multivariable Linear Regression of Factors Associated with Beverage Consumption Subgroup Analysis by Age Group. 8
Supplementary Figures........ 16
Supplementary Figure S1 16
Supplementary Figure S2 17

Supplementary Tables

Supplementary Table S1 Classification of Beverage Types

Main Beverage Category	Detailed Fluid Types
**Plain Water**	Bottled water (still, sparkling); Tap/filtered/boiled water
**Milk & Derivatives**	Low-fat/full-fat milk, fermented milk, yogurt milk, flavored milk, powdered milk, milk-based cocoa drinks, almond/rice milk, milk shakes
**Coffee and tea**	**Coffee:** Brewed, instant, vending machine, franchise coffee **Tea:** Homemade tea (hot/cold), herbal infusions, unsweetened packaged tea
**Sugar-Sweetened Beverages (SSB)**	Carbonated drinks (cola, soda, tonic); juice-based drinks with added sugar; functional beverages (sports/vitamin/energy drinks); ready-to-drink (RTD) with added sugar; tea & coffee (bottled tea, bottled coffee, bubble tea) with added sugar; flavored water with added sugar; sweetened packaged soy drinks
**100% Fruit Juices**	Bottled or freshly squeezed 100% fruit/vegetable juice (homemade or purchased)
**Artificially Sweetened Beverages (ASB)**	Diet/light/zero soft drinks, sugar-free packaged juices or nectars, beverages with non-nutritive sweeteners
**Alcoholic Beverages**	Beer, wine, champagne, aperitifs, spirits (vodka, rum, tequila), mixed alcoholic beverages
**Other Beverages**	Meal replacement drinks, alcohol-free beer, participant-specified beverages not listed above

Beverage classification was based on the *Chinese Beverage Classification Guideline* (GB/T 10789-2015), adapted to align with the *Chinese Dietary Guidelines* and previous epidemiological studies. Seven categories were analyzed: plain water, sugar-sweetened beverages (SSBs), artificially sweetened beverages (ASBs), 100% fruit juices, coffee and tea, alcoholic beverages, and other beverages. Milk and milk derivatives were classified as food water according to the *Chinese Dietary Guidelines* and were excluded from beverage analyses.

Supplementary Table S2. Descriptive Statistics of Daily Beverage Intake (mL/day) Stratified by Participant Characteristics

Characteristic	Water (mL/day)	Coffee/Tea (mL/day)	Fruit Juices (mL/day)	SSB (mL/day)	ASB (mL/day)	Alcoholic Beverage (mL/day)	Other Beverage (mL/day)
Overall	3198	1050 (607)	0 (50)	0 (0)	50 (150)	0 (0)	0 (0)
Gender							
Male	1437	1108 (583)	0 (36)	0 (0)	52 (171)	0 (0)	0 (0)
Female	1748	1000 (619)	0 (57)	0 (0)	47 (143)	0 (0)	0 (0)
Age Group (years)							
18–19	1499	1021 (567)	0 (43)	0 (0)	43 (136)	0 (0)	0 (0)
20–21	1401	1060 (655)	0 (49)	0 (0)	50 (150)	0 (0)	0 (0)
22–25	281	1086 (610)	0 (80)	0 (0)	75 (204)	0 (0)	0 (0)
Ethnicity							
Han	2817	1052 (605)	0 (50)	0 (0)	52 (154)	0 (0)	0 (0)
Non-Han	361	964 (628)	0 (50)	0 (0)	35 (130)	0 (0)	0 (0)
Physical Activity Level							
Low	552	1000 (623)	0 (57)	0 (0)	68 (158)	0 (0)	0 (0)
Moderate	1322	1100 (588)	0 (50)	0 (0)	57 (143)	0 (0)	0 (0)
High	1294	1021 (592)	0 (43)	0 (0)	36 (150)	0 (0)	0 (0)
City Tier							
1	398	857 (594)	0 (79)	0 (0)	100 (198)	0 (0)	0 (0)
1.5	814	979 (693)	0 (71)	0 (0)	79 (202)	0 (0)	0 (0)
2	1986	1093 (561)	0 (29)	0 (0)	24 (107)	0 (0)	0 (0)

This table presents descriptive statistics for daily beverage intake, stratified by various participant characteristics, including gender, age group, ethnicity, physical activity level, and city tier. The values are reported as medians with interquartile ranges (IQR) in parentheses, except for categorical variables like fruit juices and alcohol, where intake was reported as zero for a large portion of the sample.

Supplementary Table S3 Multivariable Linear Regression of Factors Associated with Beverage Consumption Subgroup Analysis by Sex.

Beverage Types	Variables	Male		Female	Title 4
		β (95% CI)	*p*	β (95% CI)	*p*
Plain Water (mL/day)	Age(years)	23.754[3.94, 43.568]	0.019	46.168[27.736, 64.599]	<0.001
	Ethnicity (non-Han vs. Han)	1.521[-80.985, 84.028]	0.971	-66.948[-139.06, 5.164]	0.069
	PA Level (Moderate vs. Low)	-50.81[-131.103, 29.483]	0.215	10.451[-52.543, 73.445]	0.745
	PA Level (High vs. Low)	8.674[-69.248, 86.597]	0.827	24.586[-41.68, 90.851]	0.467
	PSQI Score	-3.685[-14.335, 6.965]	0.497	-4.604[-13.882, 4.675]	0.331
	SAS Score	-4.027[-8.406, 0.352]	0.071	0.986[-3.469, 5.441]	0.664
	SDS Score	2.766[-1.574, 7.107]	0.211	1.543[-2.711, 5.798]	0.477
	Region Temperature (℃)	42.745[-80.759, 166.249]	0.272	20.306[-80.223, 120.834]	0.479
	Region Temperature (%)	-3.099[-25.043, 18.844]	0.600	-0.361[-18.168, 17.446]	0.939
	Socioeconomical Tier (1.5)	203.403[-460.646, 867.453]	0.327	156.74[-411.308, 724.789]	0.353
	Socioeconomical Tier (2)	307.83[-420.516, 1036.175]	0.214	376.376[-240.67, 993.422]	0.119
Coffee and Tea (mL/day)	Age(years)	9.389[3.782, 14.995]	0.001	-183.297[-452.095, 85.502]	0.120
	Ethnicity (non-Han vs. Han)	-1.176[-24.536, 22.183]	0.921	5.306[1.223, 9.389]	0.011
	PA Level (Moderate vs. Low)	-4.682[-27.412, 18.048]	0.686	6.019[-9.984, 22.022]	0.461
	PA Level (High vs. Low)	6.099[-15.965, 28.163]	0.588	-4.368[-18.347, 9.61]	0.540
	PSQI Score	1.036[-1.928, 3.999]	0.493	2.452[-12.259, 17.163]	0.744
	SAS Score	0.952[-0.287, 2.192]	0.132	1.52[-0.529, 3.569]	0.146
	SDS Score	0.032[-1.196, 1.26]	0.959	0.556[-0.434, 1.545]	0.271
	Region Temperature (℃)	9.643[-6.057, 25.343]	0.124	-0.925[-1.868, 0.019]	0.055
	Region Temperature (%)	-1.491[-4.324, 1.343]	0.157	7.748[-7.372, 22.868]	0.157
	Socioeconomical Tier (1.5)	17.232[-62.542, 97.005]	0.514	-1.21[-3.893, 1.473]	0.189
	Socioeconomical Tier (2)	26.302[-62, 114.604]	0.376	15.599[-71.867, 103.065]	0.504
100% Fruit Juices (mL/day)	Age(years)	-1.882[-4.276, 0.512]	0.123	2.171[-0.635, 4.977]	0.129
	Ethnicity (non-Han vs. Han)	-6.775[-16.744, 3.194]	0.183	12.357[1.381, 23.333]	0.027
	PA Level (Moderate vs. Low)	-3.352[-13.054, 6.349]	0.498	-2.245[-11.834, 7.343]	0.646
	PA Level (High vs. Low)	-1.493[-10.907, 7.922]	0.756	-5.111[-15.197, 4.976]	0.320

	PSQI Score	-0.502[-1.789, 0.786]	0.445	0.613[-0.799, 2.026]	0.394
	SAS Score	0.728[0.199, 1.257]	0.007	0.043[-0.636, 0.721]	0.902
	SDS Score	-0.132[-0.657, 0.392]	0.620	0.157[-0.491, 0.804]	0.635
	Region Temperature (℃)	-7.539[-22.769, 7.692]	0.168	-11.155[-26.856, 4.546]	0.093
	Region Temperature (%)	1.439[-1.266, 4.143]	0.150	2.262[-0.519, 5.043]	0.073
	Socioeconomical Tier (1.5)	-20.923[-103.105, 61.259]	0.402	-35.577[-124.228, 53.074]	0.224
	Socioeconomical Tier (2)	-20.61[-110.648, 69.428]	0.439	-27.584[-123.876, 68.707]	0.339
SSB (mL/day)	Age(years)	-1.495[-8.418, 5.428]	0.672	2.351[-2.804, 7.506]	0.371
	Ethnicity (non-Han vs. Han)	-7.284[-36.127, 21.559]	0.620	-12.487[-32.67, 7.696]	0.225
	PA Level (Moderate vs. Low)	7.96[-20.106, 36.025]	0.578	-14.216[-31.847, 3.414]	0.114
	PA Level (High vs. Low)	7.785[-19.458, 35.028]	0.575	-10.892[-29.442, 7.658]	0.250
	PSQI Score	3.319[-0.347, 6.984]	0.076	2.232[-0.36, 4.823]	0.091
	SAS Score	-0.577[-2.107, 0.954]	0.460	0.038[-1.21, 1.285]	0.953
	SDS Score	0.152[-1.364, 1.669]	0.844	1.35[0.16, 2.541]	0.026
	Region Temperature (℃)	12.621[-7.683, 32.925]	0.121	11.992[-10.533, 34.517]	0.151
	Region Temperature (%)	-2.44[-6.098, 1.218]	0.105	-1.7[-5.692, 2.293]	0.211
	Socioeconomical Tier (1.5)	26.269[-77.279, 129.818]	0.447	4.728[-123.667, 133.124]	0.887
	Socioeconomical Tier (2)	-51.334[-165.971, 63.304]	0.221	-9.213[-148.772, 130.346]	0.800
ASB (mL/day)	Age(years)	-0.419[-2.433, 1.595]	0.683	0.052[-0.222, 0.327]	0.710
	Ethnicity (non-Han vs. Han)	-4.651[-13.048, 3.746]	0.277	0.363[-0.725, 1.45]	0.513
	PA Level (Moderate vs. Low)	0.455[-7.715, 8.624]	0.913	-0.724[-1.674, 0.226]	0.135
	PA Level (High vs. Low)	0.231[-7.701, 8.162]	0.955	-0.407[-1.409, 0.595]	0.425
	PSQI Score	1.156[0.104, 2.207]	0.031	0.141[0.005, 0.276]	0.042
	SAS Score	-0.227[-0.673, 0.218]	0.317	-0.082[-0.149, -0.014]	0.018
	SDS Score	0.027[-0.414, 0.468]	0.904	0.051[-0.013, 0.115]	0.117
	Region Temperature (℃)	0.485[-4.614, 5.584]	0.707	0.166[-0.07, 0.402]	0.167
	Region Temperature (%)	0.057[-0.892, 1.005]	0.800	0.011[-0.031, 0.052]	0.620
	Socioeconomical Tier (1.5)	-4.655[-28.754, 19.444]	0.542	0.053[-1.193, 1.298]	0.934
	Socioeconomical Tier (2)	-13.266[-40.047, 13.516]	0.183	0.155[-1.191, 1.502]	0.821
	Age(years)	3.902[2.108, 5.696]	<0.001	0.137[-0.244, 0.519]	0.481

Alcoholic Beverages (mL/day)	Ethnicity (non-Han vs. Han)	9.815[2.341, 17.289]	0.010	-0.498[-2, 1.003]	0.515
	PA Level (Moderate vs. Low)	-1.369[-8.641, 5.903]	0.712	-1.029[-2.34, 0.283]	0.124
	PA Level (High vs. Low)	-0.654[-7.713, 6.405]	0.856	-0.497[-1.879, 0.885]	0.481
	PSQI Score	-0.087[-1.037, 0.863]	0.857	-0.042[-0.233, 0.148]	0.662
	SAS Score	-0.302[-0.699, 0.094]	0.135	-0.021[-0.114, 0.072]	0.663
	SDS Score	0.219[-0.174, 0.612]	0.274	0.033[-0.055, 0.122]	0.460
	Region Temperature (℃)	1.336[-4.546, 7.218]	0.422	0.388[-0.47, 1.247]	0.202
	Region Temperature (%)	-0.162[-1.226, 0.901]	0.561	-0.082[-0.234, 0.07]	0.153
	Socioeconomical Tier (1.5)	-2.484[-31.952, 26.984]	0.774	1.894[-3.286, 7.074]	0.248
	Socioeconomical Tier (2)	-3.359[-36.149, 29.432]	0.719	1.174[-4.47, 6.817]	0.451
Other Beverages (mL/day)	Age(years)	-0.294[-2.702, 2.115]	0.811	0.727[-1.657, 3.11]	0.550
	Ethnicity (non-Han vs. Han)	-5.791[-15.82, 4.239]	0.258	-1.069[-10.385, 8.246]	0.822
	PA Level (Moderate vs. Low)	5.94[-3.821, 15.7]	0.233	4.749[-3.389, 12.887]	0.253
	PA Level (High vs. Low)	8.963[-0.509, 18.435]	0.064	7.428[-1.131, 15.986]	0.089
	PSQI Score	0.057[-1.238, 1.353]	0.931	-0.42[-1.622, 0.781]	0.493
	SAS Score	0.211[-0.322, 0.743]	0.438	-0.042[-0.618, 0.533]	0.885
	SDS Score	0.034[-0.494, 0.562]	0.900	-0.216[-0.766, 0.334]	0.441
	Region Temperature a(℃)	-5.18[-21.051, 10.691]	0.295	-6.081[-28.627, 16.464]	0.365
	Region Temperature (%)	0.389[-2.429, 3.207]	0.611	0.767[-3.223, 4.756]	0.495
	Socioeconomical Tier (1.5)	-22.754[-108.445, 62.936]	0.382	-19.551[-145.549, 106.447]	0.571
	Socioeconomical Tier (2)	-25.717[-119.583, 68.15]	0.366	-34.704[-171.447, 102.039]	0.386

Values are presented as β (95% CI) with corresponding P-values from multivariable linear regression models. Beverage categories included plain water, coffee/tea, fruit juices, sugar-sweetened beverages (SSB), artificially sweetened beverages (ASB), alcoholic beverages, and other beverages. Independent variables included age (continuous), ethnicity (non-Han vs Han), physical activity (PA) level (moderate and high, reference = low), sleep quality score (PSQI_S), anxiety score (SAS), depression score (SDS), regional average temperature (°C) and humidity (%), and socioeconomic tier (1.5 and 2, reference = 1). All models were adjusted for the above covariates to minimize confounding. Statistical significance is denoted at $p < 0.05$.

Supplementary Table S4. Multivariable Linear Regression of Factors Associated with Beverage Consumption Subgroup Analysis by Age Group.

Beverage Types	Variables	18-19 years		20-21 years		22-25 years	
		**β (95% CI)**	***p***	**β (95% CI)**	**P**	**β (95% CI)**	***p***
Plain Water (mL/day)	Gender (female vs male)	-116.616[-166.027, -67.205]	<0.001	-125.114[-180.271, -69.957]	<0.001	-34.999[-159.818, 89.82]	0.581
	Ethnicity (non-Han vs. Han)	-74.196[-153.618, 5.226]	0.067	-3.653[-87.948, 80.641]	0.932	-23.119[-189.386, 143.149]	0.784
	PA Level (Moderate vs. Low)	-18.332[-90.119, 53.455]	0.617	25.573[-50.752, 101.898]	0.511	-116.767[-290.368, 56.833]	0.187
	PA Level (High vs. Low)	3.837[-67.297, 74.97]	0.916	39.715[-40.505, 119.935]	0.332	74.632[-94.348, 243.611]	0.385
	a	-2.995[-12.776, 6.787]	0.548	-8.645[-19.499, 2.209]	0.118	-7.007[-31.349, 17.334]	0.571
	SAS Score	-1.053[-5.248, 3.143]	0.623	0.501[-4.662, 5.663]	0.849	-15.587[-27.087, -4.087]	0.008
	SDS Score	0.493[-3.613, 4.599]	0.814	2.838[-2.045, 7.722]	0.254	8.265[-3.564, 20.094]	0.17
	Region Temperature (℃)	15.554[-81.995, 113.103]	0.546	43.165[-20.305, 106.635]	0.098	5.226[-271.786, 282.239]	0.95
	Region Temperature (%)	0.906[-15.761, 17.573]	0.835	-3.643[-16.601, 9.314]	0.296	6.377[-44.317, 57.07]	0.668
	Socioeconomical Tier (1.5)	188.344[-338.191, 714.879]	0.259	195.094[-181.528, 571.715]	0.143	72.107[-1707.409, 1851.623]	0.871
	Socioeconomical Tier (2)	293.156[-275.648, 861.961]	0.155	370.906[-39.32, 781.133]	0.059	409.912[-1486.399, 2306.222]	0.441
Coffee and Tea (mL/day)	Gender (female vs male)	10.303[-0.272, 20.878]	0.056	-1.914[-16.183, 12.354]	0.792	2.505[-42.256, 47.267]	0.912
	Ethnicity (non-Han vs. Han)	0.091[-16.893, 17.075]	0.992	17.038[-4.777, 38.853]	0.126	-27.564[-86.896, 31.768]	0.361
	PA Level (Moderate vs. Low)	-2.718[-18.09, 12.654]	0.729	-11.271[-31.009, 8.468]	0.263	21.739[-40.55, 84.029]	0.493
	PA Level (High vs. Low)	-3.427[-18.624, 11.77]	0.658	-2.317[-23.08, 18.446]	0.827	45.183[-14.665, 105.031]	0.138
	PSQI Score	0.878[-1.184, 2.939]	0.403	1.201[-1.595, 3.997]	0.399	7.087[-1.37, 15.543]	0.100
	SAS Score	-0.203[-1.102, 0.695]	0.657	2.121[0.786, 3.457]	0.002	0.664[-3.385, 4.712]	0.747
	SDS Score	0.003[-0.875, 0.881]	0.994	-0.389[-1.652, 0.873]	0.545	-2.303[-6.487, 1.88]	0.279
	Region Temperature (℃)	7.005[-8.148, 22.158]	0.147	10.116[-2.711, 22.942]	0.078	22.629[3.581, 41.678]	0.020
	Region Humanity (%)	-1.077[-3.385, 1.232]	0.163	-1.824[-4.554, 0.907]	0.095	-3.565[-6.666, -0.464]	0.024
	Socioeconomical Tier (1.5)	21.582[-48.811, 91.976]	0.287	14.495[-62.411, 91.402]	0.489	28.133[-48.648, 104.915]	0.471
	Socioeconomical Tier (2)	10.683[-67.036, 88.401]	0.573	9.414[-74.292, 93.121]	0.663	12.166[-73.413, 97.745]	0.780
100% Fruit Juices	Gender (female vs male)	1.122[-6.961, 9.205]	0.785	7.505[1.011, 13.998]	0.024	7.427[-6.198, 21.053]	0.284
	Ethnicity (non-Han vs. Han)	6.013[-6.981, 19.006]	0.364	-1.331[-11.247, 8.584]	0.792	14.784[-3.353, 32.921]	0.110

(mL/day)	PA Level (Moderate vs. Low)	-10.344[-22.087, 1.398]	0.084	7.569[-1.422, 16.561]	0.099	-14.708[-33.673, 4.256]	0.128
	PA Level (High vs. Low)	-6.92[-18.559, 4.719]	0.244	4.057[-5.377, 13.491]	0.399	-16.837[-35.207, 1.533]	0.072
	PSQI Score	-0.506[-2.109, 1.097]	0.536	0.568[-0.722, 1.857]	0.388	1.211[-1.418, 3.841]	0.365
	SAS Score	0.417[-0.269, 1.103]	0.233	0.481[-0.126, 1.089]	0.12	0.178[-1.071, 1.426]	0.779
	SDS Score	-0.102[-0.774, 0.569]	0.765	0.273[-0.303, 0.848]	0.353	-1.001[-2.289, 0.286]	0.127
	Region Temperature (℃)	-9.241[-26.265, 7.783]	0.147	-9.79[-23.366, 3.786]	0.09	-7.732[-32.339, 16.875]	0.262
	Region Temperature (%)	1.839[-1.105, 4.782]	0.119	1.929[-0.546, 4.404]	0.077	1.479[-3.601, 6.559]	0.247
	Socioeconomical Tier (1.5)	-23.355[-116.252, 69.542]	0.406	-29.931[-106.672, 46.809]	0.229	-37.757[-311.55, 236.035]	0.329
	Socioeconomical Tier (2)	-21.374[-121.817, 79.069]	0.472	-24.961[-108.332, 58.41]	0.318	-31.588[-304.072, 240.897]	0.403
SSB (mL/day)	Gender (female vs male)	-19.921[-35.849, -3.994]	0.014	-24.282[-40.689, -7.875]	0.004	-17.83[-60.661, 25]	0.413
	Ethnicity (non-Han vs. Han)	-16.159[-41.762, 9.444]	0.216	-13.017[-38.075, 12.041]	0.308	13.233[-43.541, 70.006]	0.647
	PA Level (Moderate vs. Low)	-14.797[-37.937, 8.342]	0.21	-5.698[-28.413, 17.016]	0.623	31.666[-27.938, 91.269]	0.296
	PA Level (High vs. Low)	-17.656[-40.589, 5.277]	0.131	2.213[-21.63, 26.055]	0.856	54.8[-2.467, 112.068]	0.061
	PSQI Score	2.23[-0.926, 5.387]	0.166	2.741[-0.51, 5.992]	0.098	5.463[-2.629, 13.555]	0.185
	SAS Score	-0.563[-1.916, 0.789]	0.414	-0.29[-1.825, 1.245]	0.711	1.405[-2.469, 5.279]	0.476
	SDS Score	0.568[-0.756, 1.891]	0.4	1.007[-0.446, 2.461]	0.174	0.51[-3.493, 4.513]	0.802
	Region Temperature (℃)	17.54[-14.647, 49.728]	0.143	6.875[-19.135, 32.884]	0.381	31.341[13.114, 49.568]	0.001
	Region Temperature (%)	-3.081[-8.623, 2.461]	0.142	-1.052[-5.884, 3.779]	0.436	-5.352[-8.319, -2.385]	<0.001
	Socioeconomical Tier (1.5)	60.259[-114.704, 235.221]	0.283	-30.846[-179.197, 117.506]	0.463	109.105[35.635, 182.576]	0.004
	Socioeconomical Tier (2)	11.106[-178.005, 200.217]	0.829	-60.925[-222.201, 100.351]	0.243	55.997[-25.891, 137.885]	0.179
ASB (mL/day)	Gender (female vs male)	-7.47[-22.819, 7.879]	0.339	-5.458[-9.806, -1.109]	0.014	-0.404[-2.292, 1.483]	0.674
	Ethnicity (non-Han vs. Han)	6.199[-14.241, 26.638]	0.551	-1.834[-8.49, 4.822]	0.589	-1.472[-3.985, 1.042]	0.250
	PA Level (Moderate vs. Low)	-2.987[-6.159, 0.185]	0.065	-5.458[-9.806, -1.109]	0.014	-0.404[-2.292, 1.483]	0.674
	PA Level (High vs. Low)	-2.286[-7.383, 2.812]	0.379	-1.834[-8.49, 4.822]	0.589	-1.472[-3.985, 1.042]	0.25
	PSQI Score	-2.731[-7.341, 1.878]	0.245	2.078[-3.932, 8.088]	0.498	0.259[-2.368, 2.886]	0.846
	SAS Score	-0.05[-4.614, 4.513]	0.983	-0.224[-6.56, 6.113]	0.945	-0.563[-3.111, 1.985]	0.664
	SDS Score	0.275[-0.349, 0.9]	0.387	0.892[0.052, 1.733]	0.037	0.075[-0.29, 0.44]	0.687
	Region Temperature (℃)	-0.263[-0.532, 0.007]	0.056	-0.01[-0.417, 0.397]	0.962	-0.015[-0.188, 0.158]	0.866
	Region Temperature (%)	0.088[-0.176, 0.352]	0.513	-0.008[-0.392, 0.376]	0.968	0.051[-0.128, 0.229]	0.578
	Socioeconomical Tier (1.5)	0.561[-4.467, 5.588]	0.645	0.264[-1.182, 1.71]	0.72	0.004[-2.211, 2.219]	0.996

	Socioeconomical Tier (2)	0.021[-0.811, 0.853]	0.92	0.043[-0.188, 0.274]	0.715	0.029[-0.379, 0.436]	0.829
Alcoholic Beverages (mL/day)	Gender (female vs male)	-0.122[-1.125, 0.881]	0.812	-5.715[-9.716, -1.713]	0.005	-16.388[-31.595, -1.181]	0.035
	Ethnicity (non-Han vs. Han)	0.103[-1.509, 1.715]	0.9	6.695[0.581, 12.809]	0.032	7.282[-12.875, 27.438]	0.478
	PA Level (Moderate vs. Low)	0.434[-1.023, 1.892]	0.559	-0.527[-6.065, 5.011]	0.852	-7.138[-28.3, 14.023]	0.507
	PA Level (High vs. Low)	0.773[-0.671, 2.216]	0.294	0.882[-4.936, 6.701]	0.766	-8.337[-28.669, 11.995]	0.420
	PSQI Score	-0.101[-0.299, 0.097]	0.316	0.125[-0.664, 0.914]	0.756	-0.604[-3.477, 2.269]	0.679
	SAS Score	-0.09[-0.175, -0.005]	0.038	-0.284[-0.658, 0.091]	0.138	-0.602[-1.977, 0.774]	0.390
	SDS Score	0.117[0.034, 0.201]	0.006	0.226[-0.129, 0.58]	0.211	0.077[-1.344, 1.499]	0.915
	Region Temperature (℃)	0.615[-0.752, 1.981]	0.202	1.265[-3.397, 5.928]	0.375	4.536[-1.935, 11.008]	0.169
	Region Temperature (%)	-0.147[-0.378, 0.085]	0.121	-0.135[-1.043, 0.774]	0.565	-0.874[-1.927, 0.18]	0.104
	Socioeconomical Tier (1.5)	2.271[-5.017, 9.56]	0.343	-1.752[-28.885, 25.382]	0.804	16.14[-9.945, 42.225]	0.224
	Socioeconomical Tier (2)	2.366[-5.524, 10.257]	0.359	-3.775[-33.303, 25.752]	0.631	14.615[-14.459, 43.688]	0.323
Other Beverages (mL/day)	Gender (female vs male)	-3.492[-10.044, 3.059]	0.296	-2.199[-8.805, 4.406]	0.514	-7.47[-22.819, 7.879]	0.339
	Ethnicity (non-Han vs. Han)	-5.848[-16.381, 4.684]	0.276	-0.381[-10.464, 9.702]	0.941	6.199[-14.241, 26.638]	0.551
	PA Level (Moderate vs. Low)	4.298[-5.219, 13.815]	0.376	4.108[-5.039, 13.254]	0.378	14.403[-6.955, 35.761]	0.185
	PA Level (High vs. Low)	10.744[1.308, 20.18]	0.026	2.618[-6.974, 12.211]	0.592	14.6[-6.128, 35.328]	0.167
	PSQI Score	-0.384[-1.686, 0.918]	0.563	-0.072[-1.387, 1.243]	0.914	1.606[-1.368, 4.58]	0.289
	SAS Score	0.408[-0.148, 0.964]	0.151	-0.18[-0.798, 0.438]	0.567	-0.314[-1.724, 1.095]	0.661
	SDS Score	-0.042[-0.586, 0.503]	0.881	-0.029[-0.615, 0.556]	0.922	-0.695[-2.147, 0.758]	0.347
	Region Temperature (℃)	-5.778[-24.576, 13.019]	0.311	-4.858[-25.833, 16.117]	0.426	-5.461[-25.077, 14.155]	0.437
	Region Temperature (%)	0.635[-2.639, 3.91]	0.49	0.342[-3.411, 4.095]	0.731	0.531[-3.085, 4.146]	0.649
	Socioeconomical Tier (1.5)	-15.673[-118.954, 87.609]	0.578	-15.299[-132.659, 102.061]	0.631	-52.756[-185.424, 79.911]	0.221
	Socioeconomical Tier (2)	-27.556[-139.298, 84.186]	0.398	-23.499[-150.886, 103.889]	0.51	-63.969[-204.01, 76.073]	0.188

Values are presented as β (95% CI) with corresponding P-values from multivariable linear regression models. Beverage categories include plain water, coffee/tea, fruit juices, sugar-sweetened beverages (SSB), artificially sweetened beverages (ASB), alcoholic beverages, and other beverages. Covariates include gender (female vs male), ethnicity (non-Han vs Han), physical activity (PA) level (moderate or high vs low), sleep quality (PSQI score), anxiety (SAS score), depression (SDS score), regional temperature (°C), regional humidity (%), and socioeconomic tier (1.5 and 2 vs 1). All models were mutually adjusted. Statistical significance is denoted at $p < 0.05$

Supplementary Table S5. Interaction Effects of Age, Sex, and Other Covariates on Beverage Consumption.

Beverage Types	Variables	β (95% CI)	*p*
Plain Water (mL/day)	Age group (20-21years vs 18-19 years)	75.881[21.573, 130.189]	0.006
	Age group (>=22 years vs 18-19 years)	120.68[29.737, 211.623]	0.009
	Gender (Female vs Male)	-111.051[-161.79, -60.312]	<0.001
	Ethnicity (non-Han vs Han)	-43.179[-97.593, 11.235]	0.12
	PA Level (Moderate vs Low)	-8.479[-58.22, 41.262]	0.738
	PA Level (High vs Low)	28.4[-22.066, 78.866]	0.27
	PSQI Score	-5.01[-12.02, 2]	0.161
	SAS Score	-1.885[-5.009, 1.239]	0.237
	SDS Score	2.283[-0.755, 5.322]	0.141
	Region Temperature (℃)	33.122[-56.624, 122.867]	0.251
	Region Temperature (%)	-2.097[-18.031, 13.838]	0.623
	Socioeconomical Tier (1.5)	192.836[-303.478, 689.151]	0.235
	Socioeconomical Tier (2)	361.158[-179.534, 901.85]	0.102
	Age group (20-21 years): Female	-14.699[-86.81, 57.411]	0.689
Coffee and Tea (mL/day)	Age group (20-21years vs 18-19 years)	9.166[-4.458, 22.79]	0.187
	Age group (>=22 years vs 18-19 years)	29.708[6.893, 52.523]	0.011
	Gender (Female vs Male)	9.913[-2.819, 22.644]	0.127
	Ethnicity (non-Han vs Han)	3.456[-10.187, 17.1]	0.619
	PA Level (Moderate vs Low)	-4.858[-17.329, 7.613]	0.445
	PA Level (High vs Low)	3[-9.661, 15.661]	0.642
	PSQI Score	1.488[-0.25, 3.225]	0.093
	SAS Score	0.738[-0.046, 1.522]	0.065
	SDS Score	-0.401[-1.162, 0.36]	0.302
	Region Temperature (℃)	9.448[-1.207, 20.102]	0.062
	Region Temperature (%)	-1.54[-3.457, 0.377]	0.074

	Socioeconomical Tier (1.5)	16.543[-41.713, 74.799]	0.354
	Socioeconomical Tier (2)	10.436[-53.639, 74.512]	0.559
100% Fruit Juices (mL/day)	Age group (20-21years vs 18-19 years)	-3.049[-10.571, 4.473]	0.427
	Age group (>=22 years vs 18-19 years)	-4.359[-16.955, 8.237]	0.497
	Gender (Female vs Male)	0.717[-6.31, 7.744]	0.841
	Ethnicity (non-Han vs Han)	3.811[-3.726, 11.348]	0.322
	PA Level (Moderate vs Low)	-2.892[-9.782, 3.998]	0.411
	PA Level (High vs Low)	-3.11[-10.1, 3.879]	0.383
	PSQI Score	0.149[-0.823, 1.122]	0.764
	SAS Score	0.382[-0.05, 0.815]	0.083
	SDS Score	-0.02[-0.441, 0.401]	0.925
	Region Temperature (℃)	-9.565[-25.66, 6.53]	0.125
	Region Temperature (%)	1.904[-0.948, 4.757]	0.103
	Socioeconomical Tier (1.5)	-28.819[-118.013, 60.375]	0.301
	Socioeconomical Tier (2)	-24.404[-121.396, 72.588]	0.393
	Age group (20-21 years): Female	6.599[-3.389, 16.587]	0.195
	Age group (22-25 years): Female	11.803[-5.605, 29.211]	0.184
SSB (mL/day)	Age group (20-21years vs 18-19 years)	-12.796[-29.767, 4.174]	0.139
	Age group (>=22 years vs 18-19 years)	6.278[-22.14, 34.695]	0.665
	Gender (Female vs Male)	-21.806[-37.661, -5.951]	0.007
	Ethnicity (non-Han vs Han)	-11.415[-28.418, 5.588]	0.188
	PA Level (Moderate vs Low)	-5.112[-20.655, 10.43]	0.519
	PA Level (High vs Low)	-1.989[-17.758, 13.781]	0.805
	PSQI Score	2.677[0.488, 4.866]	0.017
	SAS Score	-0.43[-1.407, 0.546]	0.387
	SDS Score	0.757[-0.193, 1.706]	0.118
	Region Temperature (℃)	13.039[-12.164, 38.242]	0.157
	Region Temperature (%)	-2.16[-6.637, 2.318]	0.174

	Socioeconomical Tier (1.5)	17.877[-121.418, 157.171]	0.641
	Socioeconomical Tier (2)	-23.414[-175.242, 128.414]	0.578
	Age group (20-21 years): Female	-2.75[-25.283, 19.783]	0.811
	Age group (22-25 years): Female	-11.364[-50.638, 27.91]	0.571
ASB (mL/day)	Age group (20-21years vs 18-19 years)	0.737[-3.02, 4.494]	0.700
	Age group (>=22 years vs 18-19 years)	-3.594[-9.885, 2.698]	0.263
	Gender (Female vs Male)	-3.78[-7.291, -0.268]	0.035
	Ethnicity (non-Han vs Han)	-1.916[-5.677, 1.844]	0.318
	PA Level (Moderate vs Low)	-0.232[-3.669, 3.206]	0.895
	PA Level (High vs Low)	0.102[-3.389, 3.593]	0.954
	PSQI Score	0.524[0.05, 0.998]	0.03
	SAS Score	-0.157[-0.373, 0.059]	0.154
	SDS Score	0.043[-0.167, 0.253]	0.687
	Region Temperature (℃)	0.305[-2.014, 2.623]	0.624
	Region Temperature (%)	0.037[-0.386, 0.46]	0.728
	Socioeconomical Tier (1.5)	-0.433[-13.019, 12.153]	0.897
	Socioeconomical Tier (2)	-4.489[-18.404, 9.425]	0.297
	Age group (20-21 years): Female	-0.91[-5.897, 4.078]	0.721
	Age group (22-25 years): Female	1.915[-6.782, 10.611]	0.666
Alcoholic Beverages (mL/day)	Age group (20-21years vs 18-19 years)	4.732[1.321, 8.143]	0.007
	Age group (>=22 years vs 18-19 years)	14.953[9.241, 20.666]	<0.001
	Gender (Female vs Male)	-0.106[-3.293, 3.082]	0.948
	Ethnicity (non-Han vs Han)	4.041[0.625, 7.458]	0.02
	PA Level (Moderate vs Low)	-0.974[-4.097, 2.149]	0.541
	PA Level (High vs Low)	-0.34[-3.51, 2.83]	0.833
	PSQI Score	-0.063[-0.5, 0.374]	0.778
	SAS Score	-0.19[-0.387, 0.006]	0.057
	SDS Score	0.155[-0.036, 0.346]	0.111

	Region Temperature (℃)	0.989[-2.256, 4.234]	0.323
	Region Temperature (%)	-0.158[-0.738, 0.422]	0.361
	Socioeconomical Tier (1.5)	0.996[-16.825, 18.816]	0.836
	Socioeconomical Tier (2)	0.114[-19.416, 19.644]	0.982
	Age group (20-21 years): Female	-5.651[-10.18, -1.123]	0.014
	Age group (22-25 years): Female	-16.442[-24.336, -8.547]	<0.001
Other Beverages (mL/day)	Age group (20-21years vs 18-19 years)	-0.646[-7.463, 6.17]	0.852
	Age group (>=22 years vs 18-19 years)	0.77[-10.644, 12.184]	0.895
	Gender (Female vs Male)	-3.774[-10.142, 2.594]	0.245
	Ethnicity (non-Han vs Han)	-2.255[-9.085, 4.575]	0.518
	PA Level (Moderate vs Low)	4.951[-1.293, 11.194]	0.120
	PA Level (High vs Low)	8.018[1.684, 14.352]	0.013
	PSQI Score	-0.109[-0.991, 0.773]	0.809
	SAS Score	0.085[-0.307, 0.477]	0.671
	SDS Score	-0.081[-0.462, 0.301]	0.678
	Region Temperature (℃)	-5.785[-24.761, 13.19]	0.319
	Region Temperature (%)	0.6[-2.759, 3.96]	0.521
	Socioeconomical Tier (1.5)	-21.029[-126.297, 84.239]	0.480
	Socioeconomical Tier (2)	-31.085[-145.448, 83.277]	0.362
	Age group (20-21 years): Female	1.409[-7.642, 10.46]	0.760
	Age group (22-25 years): Female	3.557[-12.217, 19.332]	0.658

This table presents the beta coefficients (β) with 95% confidence intervals (CI) and p-values from a multivariable linear regression model examining factors associated with daily beverage intake (in milliliters or equivalent units, assumed based on context). The model includes main effects for age group (reference: 18-19 years), gender (reference: male), ethnicity (reference: Han), physical activity (PA) level (reference: low), Pittsburgh Sleep Quality Index (PSQI) score, Self-Rating Anxiety Scale (SAS) score, Self-Rating Depression Scale (SDS) score, regional average temperature (°C), regional average humidity (%), and Socioeconomic tier (reference: tier 1 = first-tier cities; tier 1.5 = emerging first-tier cities; tier 2 = second-tier cities).Interaction terms are included for age group by gender (e.g., Age group (20-21 years): Female). Beverage types analyzed include Plain Water, Coffee and Tea, Fruit Juices, Sugar-Sweetened Beverages (SSB),

Artificially Sweetened Beverages (ASB), Alcoholic Beverages, and Other Beverages. Positive β values indicate higher intake associated with the variable, while negative values indicate lower intake. Statistical significance is denoted at $p < 0.05$. All analyses adjust for potential confounders listed.

Supplementary Figures

Supplementary Figure S1

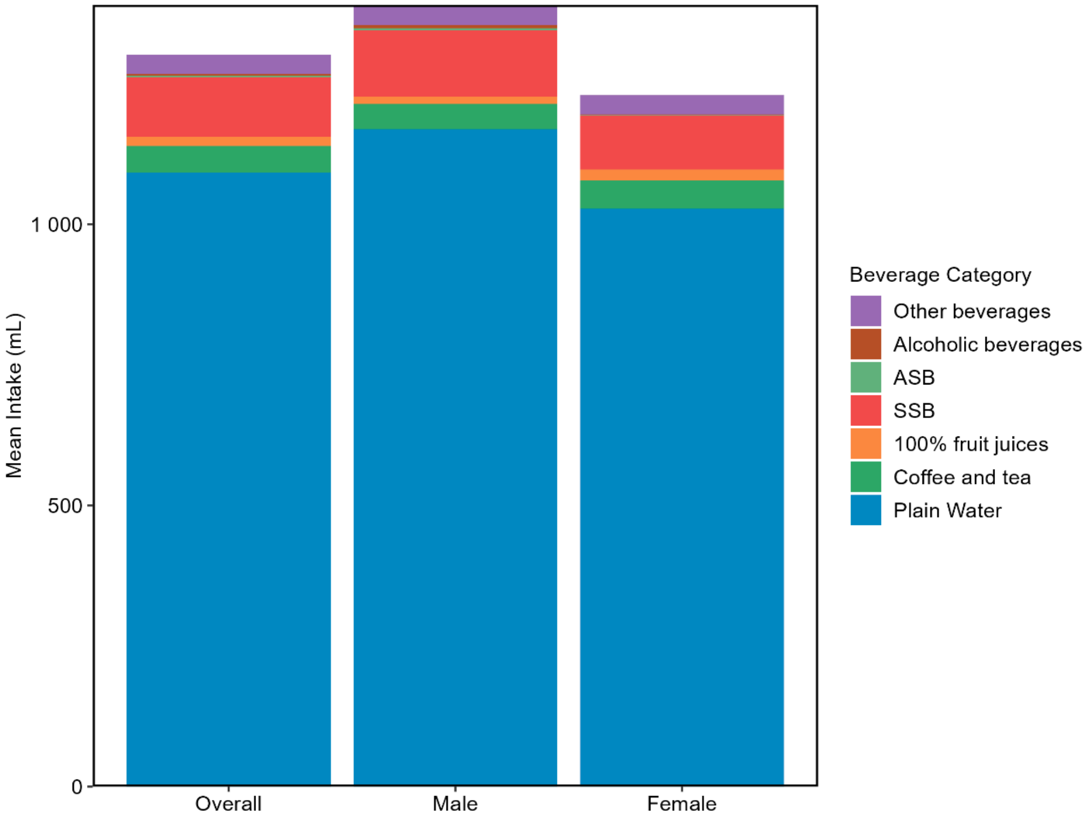

Supplementary Figure S1. Mean beverage intake (DFI) composition (mL/day) by gender (including overall). The chart displays the mean daily intake of various beverage categories for the overall population, males, and females. The total height of each bar represents the mean total DFI, and the colored segments represent the mean intake (mL/day) of each specific beverage category. Abbreviations: DFI, Drinking Fluid Intake; SSB, Sugar-Sweetened Beverages; ASB, Artificially Sweetened Beverages.

Supplementary Figure S2

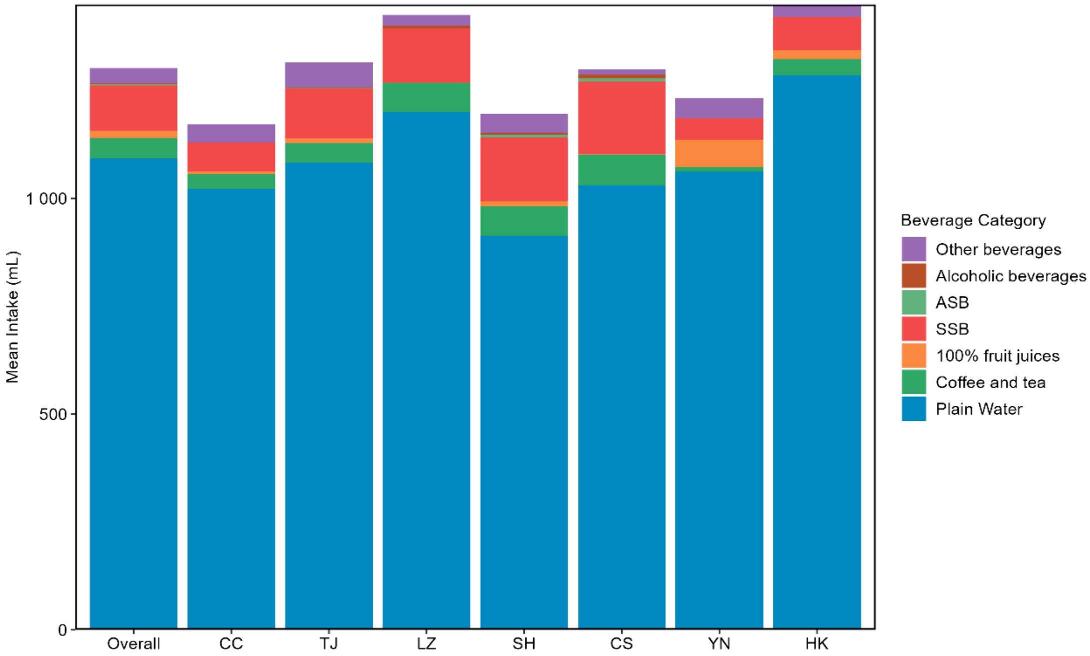

Supplementary Figure S2. Mean beverage intake (DFI) composition (mL/day) by region (including overall). The chart displays the mean daily intake of various beverage categories for the overall population and across seven different regions (CC, TJ, LZ, SH, CS, YN, HK). The total height of each bar represents the mean total DFI, and the colored segments represent the mean intake (mL/day) of each specific beverage category. Abbreviations: DFI, Drinking Fluid Intake; SSB, Sugar-Sweetened Beverages; ASB, Artificially Sweetened Beverages.
